# Influence of knot strength on the mechanical performance of a biodegradable gillnet

**DOI:** 10.1038/s41598-024-66474-3

**Published:** 2024-07-04

**Authors:** Louis Le Gué, Esther Savina, Mael Arhant, Peter Davies, Nicolas Dumergue, Benoit Vincent

**Affiliations:** 1 Ifremer, RDT Research and Technological Development, 29280 Plouzané, France; 2 Institut Agro, IFREMER, INRAE, DECOD, 56325 Lorient, France; 3 Section for Fisheries Technology, DTU Aqua, 9850 Hirtshals, Denmark

**Keywords:** Materials science, Ocean sciences

## Abstract

Ghost fishing is a global issue that can be addressed using fishing gear materials that do not persist in the marine environment. However, for these alternatives to be widely adopted, they must meet the same mechanical specifications as current commercial materials while degrading without any negative impact. The objective of this study was to compare a conventional gillnet made of polyamide 6 (PA6) with an alternative made of poly(butylene succinate-co-adipate-co-terephthalate) (PBSAT) at three different scales: monofilament, knot, and net. While the PBSAT monofilament’s strength was half that of the conventional PA6 net, knot and net losses were even more significant. This indicates a greater sensitivity of the material to the knot. Since the results between the knot and net scales were coherent, testing whole net panels is not necessary. Studying the curvature and the behaviour of the knot revealed its complex geometry and mechanical behaviour. Testing the weaver’s knot is a good indicator for studying the relevance of an alternative to conventional fishing gear materials. This should be considered when developing biodegradable nets in order to reduce ghost fishing at sea.

## Introduction

During the 1960s, synthetic fibres quickly replaced natural fibres, such as hemp and cotton, commonly used for making fishing gears^1,2^. Indeed, polymeric fibres are more resistant, durable, and less visible. However, unlike their natural predecessors, they persist in the marine environment when abandoned or lost, which allows the gear to continue fishing. Ghost fishing is used to describe the by-catch caused by abandoned, derelict, or lost fishing gear (ADLFG)^3,4^. An animal caught in a fishing net faces severe consequences, including restricted movements, suffocation, injuries, and potential death due to starvation or predation^5,6^. While towed gears, such as trawl nets, are prone to losing fragments, passive gears, like gillnets, are more susceptible to complete loss^7^ while also presenting the highest risk for the marine environment^8^. Following an eventual ghost fishing period, ADLFG also plays a role in contributing to microplastic pollution as they break down into small plastic particles^9^. Due to the chemical stability of conventional polymers^10^, these particles then gather in marine ecosystems^11–14^. In these environments, the particles can be ingested^15^, release harmful additives^16^, and carry organic pollutants^17^.

Biodegradable polyesters have been developed to retain the benefits of conventional polymers in terms of resistance and visibility without persisting in the marine environment in case of loss. These polyesters break down faster than traditional polymers in the ocean^18^, due to ester linkages promoting chain scission through hydrolysis by water and microorganism enzymes^19–21^. Biodegradation is complete when the polymer’s molecular weight decreases enough to be mineralised by microorganisms under aerobic or anaerobic conditions^22^. Fishing gear made from these polymers could thus reduce ghost fishing by degrading faster than conventional polymers^23^, but also avoid microplastic pollution by being mineralised.

Some of those polymers have already been tested as fishing gear, with poly(butylene succinate co-adipate-co-terephthalate) (PBSAT)^24–32^ and pure polybutylene succinate (PBS) resin^33^ or blended with polybutylene adipate-co-terephthalate (PBS/PBAT)^18,27,34–36^ being the most studied polymers.

Poly(butylene succinate/adipate) (PBSA) shows improved seawater degradability^37^ and biodegradation^38^ compared to PBS and was also studied for use in demersal fisheries^39^. Recently, Park et al. (2023)^40^ incorporated adipic acid and ethylene glycol into a PBS backbone to create monofilaments of polybutylene succinate co-butylene adipate co-ethylene succinate co-ethylene adipate (PBEAS) with enhanced mechanical properties. Although the biodegradation of PBEAS in composting facilities has been investigated^41^, there is currently no research on its degradation in marine ecosystems.

Polylactic acid (PLA) has been less studied because of its known stability in seawater at natural temperatures^42^ but Yu et al. (2023)^43^ found that fishing efficiency of PLA gillnets was comparable to conventional gillnets.

While the use of those polymers in ropes has been demonstrated^18,39,44^, the widespread adoption of biodegradable polymers in nets faces a technical obstacle; the loss of fishing efficiency observed by several authors^24–26,30,34,45–47^ must be offset to be viable for commercial application^48^. Grimaldo et al. (2019)^25^ found, after conducting sea trials and mechanical testing on both conventional and biodegradable gillnets, that the difference in catching efficiency was mainly due to lower mechanical properties of the biodegradable net, rather than a degradation of the polymer during fishing operations.

Improving biodegradable polymers’ properties until they meet current material standards involves understanding fishing net structures. However, most authors only test monofilaments, meshes, or knots without specifying their nature and relevance to fishing nets. This study compares the strength of a PBSAT net with a conventional PA6 net, from individual monofilaments to double weaver’s knots and complete net panels. The current state of research and development indicates that PBSAT might not be the best solution for commercial use, but it is of interest for further investigations to better understand how these new polymers behave compared to the standard plastics. The aim of this study is thus not to compare intrinsic material properties but to explore the behaviour of both nets at various levels. Valuable insights into the mechanical behaviour of fishing nets can thus be gained, which will support further testing and therefore help developing biodegradable nets that meet the required mechanical properties.

## Methods

### Materials

Two types of material were used in the present study. A polyamide 6 (PA6) with a melting temperature of $224~^{\circ }\hbox {C}$ and melting enthalpy of 69.4 $\hbox {J.g}^{-1}$ was used as the reference material. A poly(butylene succinate-co-adipate-co-terephthalate) (PBSAT) with a melting temperature of $105~^{\circ }\hbox {C}$ and a melting enthalpy of 68.3 $\hbox {J.g}^{-1}$ was studied as the biodegradable alternative. The weight average molecular weight of PBSAT pellets was 225 $\hbox {kg.mol}^{-1}$ as given by the technical data sheet of the supplier. Materials were supplied as custom-made net panels manufactured by S-EnPol (Korea) in 2020 (Ref. No. S200205-01) to fit the requirements for a Danish commercial gillnet cod fishery. Nets dimensions were the following: 150 mm full mesh size (75 mm bar length), 30.5 meshes high, 2000 knots (150 m).

Monofilament and knot samples were randomly cut from the panels to characterise the mechanical properties at different scales. Monofilament samples could not be longer than 75 mm, as this was the mesh size. Net samples were obtained by randomly cutting 7 $\times$ 3 mesh panels from the main nets.

To avoid hydrolytic degradation of the PBSAT samples^23^, all nets were stored at low temperatures before testing ($-10~^{\circ }\hbox {C}$ at DTU, $4~^{\circ }\hbox {C}$ at Ifremer).

### Mechanical testing

Mechanical testing at each scale was performed on a 10 kN capacity test machine equipped with a 500 N load cell for monofilament and knot testing and a 10 kN load cell for net samples.

Monofilament samples were tested by clamping each end of the monofilament. Displacement speed was set at 10 $\hbox {mm.min}^{-1}$. The strain was measured by following two markers placed on the samples with a digital camera.

Knots were tested by blocking their four ends in clamps. Ends of the same monofilament were clamped together according to the ISO-1806 standard. Strain measurements were made using markers on the four ends followed during the test, thanks to a camera placed in front of the sample. Knot tightening was calculated using virtual knot displacement, defined as the mean position of the two markers on the same monofilament. The tightening of the knot corresponds to the relative increase in the distance between those two virtual points during the test.

Net tests were conducted using 8 mm diameter steel snap hooks to connect the upper and lower meshes to beams fixed on the testing machine. The displacement rate was set to 50 mm/min for all samples. The same camera used for monofilament and knot testing recorded images of net panels during testing. The strain between knots was then calculated using an image correlation script for each test.

**Figure 1:**
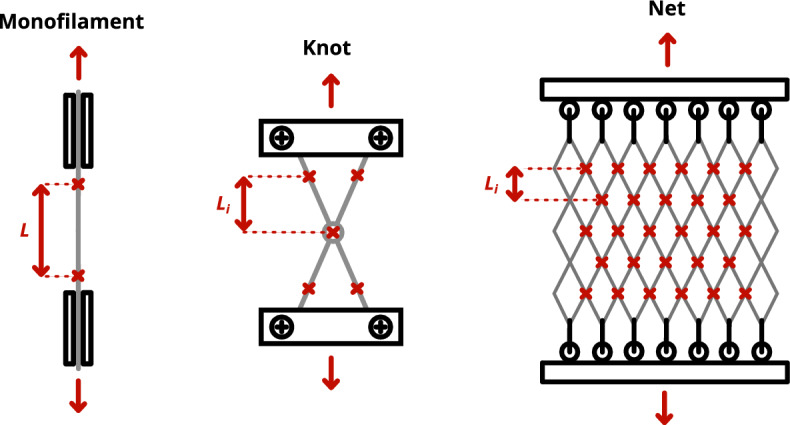
Schematic view of the method used for mechanical testing at each scale: monofilament, knot, and net.

Figure 1 summarises the different techniques used for each scale, where the red crosses represent the markers or knots followed by post-treatment. All the tests were performed on dry samples in a room maintained at 21 $^{\circ }\hbox {C}$ and 50% relative humidity.

### Steric Exclusion Chromatography (SEC)

After dissolving 20 $\mu$g samples during 10 h in 10 mL of an ethanol-stabilised chloroform solution, Steric Exclusion Chromatography (SEC) was performed at 30 $^{\circ }\hbox {C}$, with an injection volume of 100 $\mu$L, a flow rate of 1.0 $\hbox {mL.min}^{-1}$, an Agilent-DRI refractive index detector and three columns: a PS/DVB Agilent 5 $\mu$m precolumn, and two Agilent Mixed-C 5 $\mu$m columns. Calibration was performed with chloroform on a standard polystyrene.

### SEM observations

Scanning Electron Microscopy (SEM) provided surface observations on pristine samples using FEI Quanta 200 equipment. Before being placed in the microscope, samples were coated with a thin 60% gold and 40% palladium layer to avoid charging.

### X-ray tomography

X-ray tomography was performed on PBSAT knots to understand their mechanical behaviour in more detail. The tests were conducted at a resolution of 5 $\mu$m with a voltage of 60 kV and a current of 70 $\mu$A, resulting in a total power of 4.2 W. The acquisition produced 1800 images, each exposed for 2 seconds, over one hour.

### Curvature calculation

The curvatures of the knot’s monofilaments were investigated. The centerlines of both monofilaments composing the knot were first extracted from 3D scans obtained by X-ray tomographies. The extraction was based on vertical and horizontal cross-sections. Each monofilament’s cross-section was then identified, and the biggest circle to fit inside this section was used to find the centre point. Repeating this for all the main cross-sections allowed the construction of the centerlines of both monofilaments. Using triangles and their circumcircle, a Python algorithm was then used to compute the curvature change along the monofilament. The curvature $\kappa$ is defined as the inverse of the radius of the circumcircle.

### Data analysis and visualisation

All data were analysed using Python 3.9.18^49^ together with NumPy^50^ and Pandas^51^ packages. For image correlation, the Scikit-image library was used^52^ and Matplotlib was used for visualisation^53^.

## Results

### Monofilament scale

#### Mechanical testing

**Figure 2:**
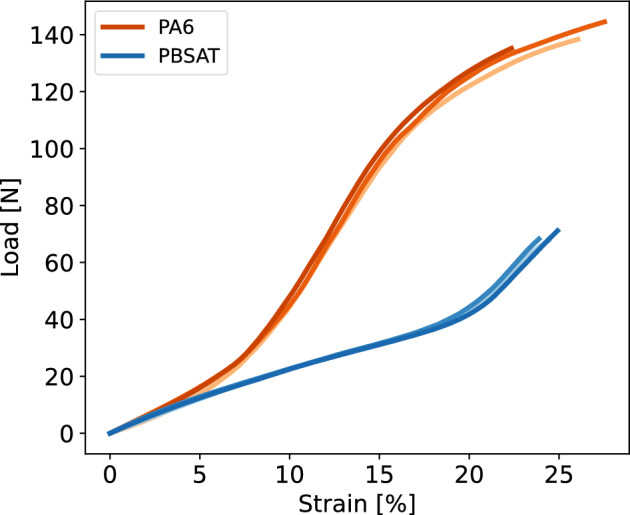
Load versus strain curves for PBSAT and PA6 monofilaments, each curve represents a tested sample.

Gillnets are made of monofilaments tied together in knots to make the final net panel. Monofilament samples were extracted from PA6 and PBSAT nets for tensile testing. Figure 2 presents the load versus strain curves for three samples of both materials after tensile testing. While a similar mean strain at break of 25% was observed for both monofilaments, the difference in load at break was significant, with respectively 72 ± 1 N (mean ± standard deviation) and 149 ± 4 N for the PBSAT and the PA6 yarns. The PBSAT monofilament exhibits a stiffness of 1.06 ± 0.09 GPa between 0 and 5% of strain, which is lower than 1.45 ± 0.13 GPa the stiffness of the PA6. The overall properties of the PBSAT monofilament are below the properties observed for the PA6, with a 51% lower load at break.

#### Molar mass measurement

In a polymer, the length of polymer chains, and thus the molar mass, plays an important role in the mechanical properties^18,54^. Steric exclusion chromatography has therefore been performed on monofilaments extracted from PBSAT nets to measure the molar mass of the polymer after it had been through the different processes to manufacture the net. Biodegradable polyesters such as PBSAT are subjected to hydrolytic degradation. The molar mass of the PBSAT samples was measured to verify the polymer integrity by comparing it with the value provided by the industrial supplier for the pellets. The weight average molecular weight measured was 79.6 ± 7.5 kg. $\hbox {mol}^{-1}$, which is −65 % lower than 225 ± 75 $\hbox {kg.mol}^{-1}$, the weight average molecular weight specified for the pellets before extrusion, net making, net stabilisation, and shipping. The number averaged molecular weight measured was 21.1 ± 1.3 kg. $\hbox {mol}^{-1}$ and the molar mass dispersity of the polymer, as determined by the ratio of the weight average molecular weight to the number average molecular weight, was $3.8 \pm 0.6$.

### Knot scale

#### Scanning electron microscopy observations

**Figure 3:**
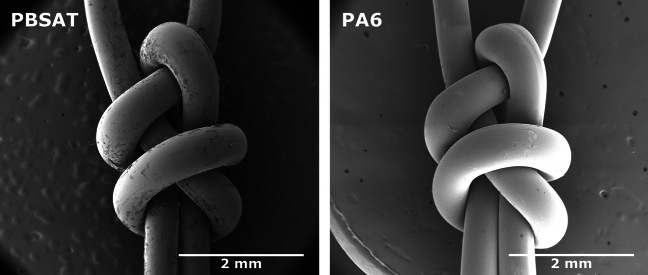
SEM observations of PBSAT and PA6 knots retrieved from gillnets.

Figure 3 presents observations of knots taken from PBSAT and PA6 nets. The double weaver’s knot allows two strands to be tied together. For both observations, the two ends of the first monofilament come from above, while the two ends of the second monofilament come from below. The second has a simple hairpin shape, while the first is woven around it with two loops. The PBSAT knot’s surface is slightly rougher and shows more impurities than its PA6 equivalent. The cross-section of the PA6 also seems to flatten at the points of curvature while the PBSAT monofilament’s cross-section maintains a relatively circular shape.

#### Mechanical testing

**Figure 4:**
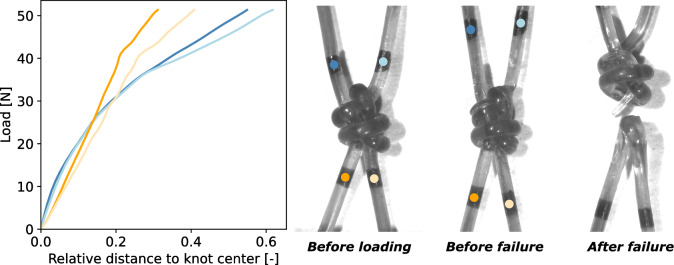
Tightening curves of the PBSAT knot, each line represents the displacement of a point that can be identified on the image by its colour.

**Figure 5:**
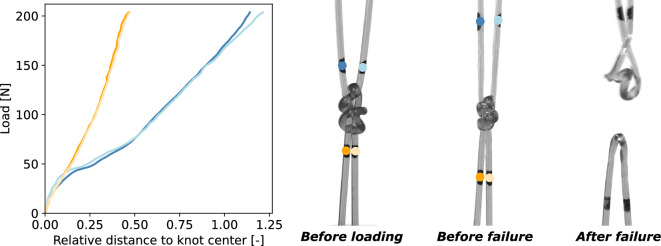
Tightening curves of the PA6 knot, each line represents the displacement of a point that can be identified on the image by its colour.

The tensile strength and mechanical characteristics of monofilaments made from PA6 and PBSAT in a double weaver’s knot configuration were examined. This involved pulling apart the two ends of each monofilament, following markers with cameras, and observing changes in the knot’s shape. Figures 4 and 5 show an example of tightening curves obtained for PBSAT and PA6 knots alongside images taken during the test. The x-axis represents the distance between each marker and the knot’s centre, with each curve corresponding to a specific marker identified by its colour. The y-axis represents the load measured by the load cell during the tightening until the failure of the knot.

For the PBSAT knot, the markers move away uniformly up to a load of 25 N. Beyond this point, there is a significant shift, with markers on the upper filament diverging further. At around 40 N the knot cracks and an inflection in all the curves is observed until the knot breaks completely in the loops of the upper monofilament. Repeating the experiment three times led to a mean load at break of 54 ± 3 N.

Markers move away uniformly until 30 N for the PA6 knot, but beyond this threshold, the markers on the upper monofilament start moving more with the increase in tension in the knot. Above 50 N, the knot is tightening, and it becomes more and more difficult for the markers to move away from the centre of the knot, with the difference between two markers of the same monofilament being very small. The rotation of the markers of the upper monofilament indicates that a twist is taking place within the knot. A divergence between the two markers of the upper monofilament is observed, indicating the beginning of the failure. After three tests on PA6 knots, an average load at break of 210 ± 6 N was found. PA6 knots are therefore four times stronger than PBSAT knots. For both knots the threshold values were similar and depended on the initial state and shape of the knot. Beyond the threshold, PA6 knots were also able to tighten more than the PBSAT knots, as noted by the compact shape of the PA6 knot before failure compared to the damaged PBSAT knot. Larger distances are also reached by markers around the knot, which induce a higher difference between markers placed on the upper monofilament and markers placed on the bottom. To sum up, PA6 knots are stronger and more capable of tightening than PBSAT knots, which results in a more asymmetric behaviour.

#### Curvature analysis

**Figure 6:**
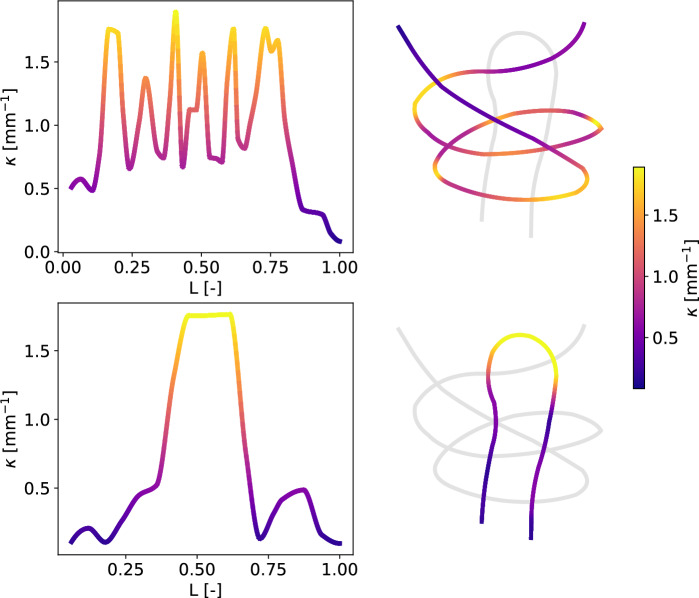
Change in curvature along the normalised length of the two monofilaments of a PBSAT double weaver’s knot.

Both monofilaments’ paths were retrieved using X-ray tomography on a PBSAT knot. The curvature $\kappa$ along each monofilament was then calculated and is presented in Fig. 6 on two separate panels, one for each monofilament. For a better understanding, a 3D view with the curvature in the concerned monofilament was added next to the corresponding panels. The curvature change in the upper monofilament is unstable, with four main peaks when the monofilament has to bypass the two ends of the bottom monofilament. The magnitude of these peaks is between 1.7 and 1.8 $\hbox {mm}^{-1}$. The two additional peaks are caused by the monofilament overlapping itself, resulting in lower magnitudes ranging from 1.4 to 1.5 $\hbox {mm}^{-1}$ as it does not fully encircle the monofilament. Apart from peaks, the curvature inside the knot is always higher than 0.5 $\hbox {mm}^{-1}$. The curvature in the second monofilament is more stable. It consists of a plateau of around 1.75 $\hbox {mm}^{-1}$ in the middle of the monofilament, which is consistent with the observed loop at this location.

### Net scale

Knots are used to manufacture a net, which is the last scale studied in this study. Nets are complex structures. Within a fishing net, a row of knots connects two monofilaments. Consequently, the same monofilament appears between two horizontal rows of knots in both the bottom and top knots. To ensure stability in the net, the orientation of the knots is alternated between each row. As a result, a monofilament passing through the net always remains in the same position within the knots it encounters.

**Figure 7:**
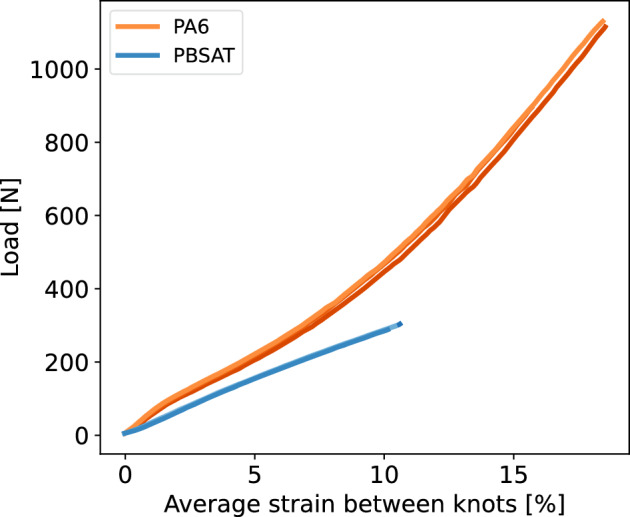
Load versus mean strain between knots curves for PBSAT and PA6 nets, each curve represents a tested sample.

Figure 7 illustrates the evolution of the applied force on the net as a function of the average strain observed between all the monitored knots for both materials studied. Three curves of the same colour (red for PA6, blue for PBSAT) represent the three samples tested for each material, with the end of the curves indicating sample failure. On average, the PBSAT net broke at 297 ± 6 N, with an average strain between the knots of 11 ± 2%. The PA6 net broke at 1061 ± 97 N with an average strain between knots of 20 ± 1%. The scatter was low for both materials. The PA6 thus appears to be nearly four times stronger than PBSAT nets, with double the average strain between knots.

**Figure 8:**
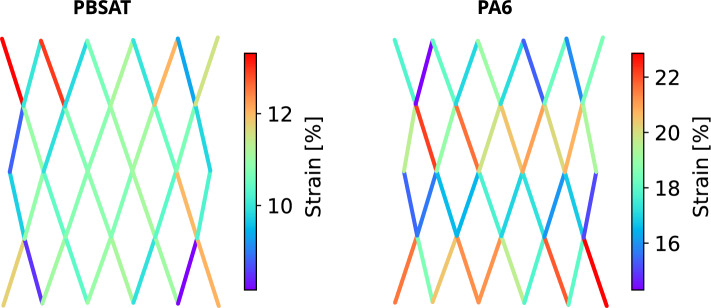
Distribution of strains between knots for a PBSAT net and a PA6 net before failure during a tensile test.

Figure 8 illustrates the distribution of the measured strain between each knot before failure for both materials. For the PBSAT net, the distribution is generally uniform, with some local maxima and minima. The measured deformations for the PA6 net exhibit a distinct pattern, with rows of high measurements alternating with rows of low measurements. However, each row corresponds to a single monofilament, so every monofilament deforms more than its neighbour, leading to an asymmetric behaviour of the meshes inside the net.

## Discussion

The PBSAT material demonstrates lower performance than PA6 at the monofilament level. However, despite lower tensile strength and strain at break, the stiffness of the monofilament remains equivalent to the PA6, which is crucial for fishing net applications as it significantly impacts selectivity and fishing efficiency. Despite having a higher stiffness, the PA6 monofilament appears to bend more effectively, as observed by the flattening of the cross-sectional area in the SEM image shown in Fig. 3. PA6 is known to plasticise when absorbing water^55^. The knot stabilisation process described by Kim et al. (2019) involves heating the net while injecting pressurised steam. This combination of higher temperature and plasticisation enables the PA6 monofilament to better adapt to curvature within the knot and accommodate stress associated with tightening. In contrast, the PBSAT monofilament is prone to chain degradation by water, called hydrolysis, a phenomenon accelerated by temperature^18,33,54^. Furthermore, the measured molar mass of the PBSAT extracted from the net is 65% lower than the pellets before processing.

This indicates that PBSAT polymer chains were degraded before reception, there are several potential causes: extrusion, net manufacturing, net stabilisation, storage between production sites, or during shipping. Hydrolysis is time and temperature dependent, so it may come from a short exposure to high temperatures (extrusion, net stabilisation) and/or long exposure to moderate temperatures (storage between extrusion and net manufacturing). Lower molar mass not only implies lower mechanical properties^18^, but also leads to faster biodegradation^56^. When developing or scaling up the production of a biodegradable net, it is therefore important to monitor the molecular weight change during the different production steps to detect and change procedures that damage the material’s polymeric structure. The impact of these lower properties on knot and net scale was then investigated.

The ISO 1806:2002 standard describes a methodology to determine the mesh breaking strength of fishing nets and has already been used to compare PA6 and PBSAT fishing nets^25^. The testing procedure involved examining two monofilaments, each passing through two knots: one knot is made of one of the tested monofilaments, and the other knot is made of both monofilaments having free ends outside the mesh. Due to the unblocked ends of both monofilaments, slippage occurred during the tests. This slippage, combined with the high displacement rate and the friction generated by the slipping monofilament within the knot, may result in an underestimation of the strength of both materials. While mesh tests are conducted on monofilaments with knots, they do not fully capture the behaviour of the monofilament inside a knot. The tests in the present study were conducted by blocking the two ends of the two monofilaments composing the knot to better understand the behaviour of the material during tightening. Furthermore, imposing a test duration time also results in different displacement rates for materials that have different mechanical properties at break, leading to different testing conditions which compromise the comparison of the results.

The difference in strength obtained between PBSAT and PA6 is even more pronounced at the knot level than at the monofilament level. The PBSAT knot experiences a loss of strength of −62%, whereas, for PA6, the loss is −30%, further widening the gap between the two materials noted at the monofilament level. However, as depicted by Figs. 4 and 5, both materials exhibited a two step tightening process. First, there is a non-plastic tightening of the knot, as seen by the uniform displacement of the four ends, followed by a plastic tightening, leading to a higher displacement of the marker on the top monofilament that is longer than the bottom one. The PA6 knot was more capable of plastically deforming, leading to an asymmetric behaviour and a more compact knot than the PBSAT, that presented cracks and failed at the beginning of plastic tightening. The non-plastic tightening may be driven by the friction coefficient of the polymer since it involves a uniform slipping of the four ends in contact with each other. The friction coefficient also plays a role in plastic tightening since both monofilaments have to pass through different loops while deforming. When looking at tensile test curves, both materials exhibited an equivalent strain at break, with the PBSAT being less stiff than the PA6. Tensile testing is, therefore, insufficient to describe the failure of the knot, and other tests should be made at the monofilament scale to understand the premature failure of the PBSAT knot. The knot exacerbates the difference in strength between the two materials due to its complexity, as evidenced by the curvature evolution in the two intertwined monofilaments forming the knot. According to theoretical principles, when a monofilament A wraps around another filament B, it develops curvature along the median line, denoted as $\kappa$. This curvature, mathematically expressed as $\kappa = 1 / (R_{A} + R_{B})$, is inversely proportional to the sum of the radii of filaments A and B. In this specific case where both filaments possess identical diameters, the equation simplifies to $\kappa = 1 / 2R$, resulting in a value of approximately 1.75 $\hbox {mm}^{-1}$, given a diameter of 0.57 mm. The observed values for both monofilaments are consistent with this theoretical framework, particularly evident in the bottom filament, which reaches a stable plateau at 1.75 $\hbox {mm}^{-1}$ when encircling the second filament. The four main peaks observed when the top monofilament bypasses the bottom monofilament’s ends are also close to this value, which thus confirms the experimental data. This is the first time that the curvature is described inside a weaver’s knot. As demonstrated by Pieranski et al. (2001)^57^, knot rupture occurs at curvature peaks. This was also observed during experiments for both materials, with a higher proportion of failures happening in the upper monofilament. The top monofilament has the area of maximum curvature and several peaks of curvature, increasing the likelihood of the monofilament breaking. However, Przybyl et al. (2009)^58^ demonstrated that the curvature evolves during tightening, which shifts the maximum curvature outside the knot for the overhand knot. Finite element models are accurate for modelling the overhand knot^59^, and could also be used to understand further the mechanical stresses in the weaver’s knot. Improved knowledge of how this knot works would help to select suitable biodegradable materials.

At the net level, the failure occurred at 297 ± 6 N for the PBSAT and 1061 ± 97 N for the PA6. When testing a sample until it fails, the failure occurs at its weakest point. In a net under tension, the weakest point is where the fewest knots absorb the force. For a 7 $\times$ 3 mesh net panel, the row with the lowest number of knots is 7. However, the outer knots do not take up any force due to slippage of the free edges of the net. This is confirmed by the shape of outer knots before the failure of the PA6 net. Therefore, the weakest point of the net is made of 5 knots, which correlates with the factor found between the knot scale strength and the net scale strength for both materials. Net strength being proportional to knot strength, it also explains the similar differences in strength of respectively 75% and 72% across the knot scale and net scales.

Observing the positions of points around the knot during tightening showed that the movement of the top monofilament is greater than that of the bottom monofilament. Each row of the net, between two rows of knots, is made of the same monofilament, which occupies the same position in the top and bottom knots it crosses. A monofilament in the position of the upper monofilament then experiences greater strain between knots, which explains the pattern observed in Fig. 8 for the PA6. The PBSAT exhibited lower tightening levels that caused the knot to break, resulting in less asymmetry between the upper and lower monofilament points within the knot. The strain distribution of the net then appears uniform, without the asymmetric pattern observed in the PA6 net. In addition to showing a clear difference between the PBSAT net and the nylon net, these tests on nets confirm that the behaviour of the knot is similar when it is tested alone to when it is tested at the scale of the net. Furthermore, since comparing materials at the knot scale would lead to the same conclusions, knot testing seems to be the easiest and most effective way to compare new biodegradable materials with conventional ones.

The study found that the molecular weight of the PBSAT monofilament decreased significantly when compared to the pellets, suggesting that the material had degraded before testing. This indicates that the mechanical properties described in the study may not accurately represent the optimal performance of a PBSAT monofilament and instead highlight its sensitivity to degradation. However, the aim of the study was to improve understanding of net structures, particularly at the knot level. Despite the limitation of using a PBSAT monofilament with lower properties, the results highlight the importance of testing the double weaver’s knot when developing a new material for netting. Furthermore, a first experimental description of the curvature in a double weaver’s knot was provided. To assess the performance of future biodegradable polymers, it is crucial to carefully handle the material during processing and storage. Monitoring the change in molecular weight during different processing stages could help evaluate the polymer’s stability.

## Conclusion

The study involved comparing and testing PBSAT and PA6 at different scales to gain insights into their differences and the functioning of net structures by analysing the weaver’s knots. PBSAT showed a 50% lower strength at the monofilament scale, which could be partially explained by the degradation of polymer chains, as indicated by molecular weight measurements. The difference in strength increased to 4 times at the knot scale and consequently at the net scale. The PBSAT knot was cracking and failing before tightening. These findings provide an additional explanation for the low fishing performance of PBSAT nets. They confirm that the lower catches observed on biodegradable nets are linked to lower mechanical properties rather than a degradation process.

Furthermore, differences in strength and overall behaviour for both materials were equivalent at both knot and net scales. Testing the whole net panels is unnecessary, and weaver’s knot testing provides a straightforward way to assess the suitability of a biodegradable material to replace a conventional material. However, fishing knots are complex, it is thus necessary to understand their mechanical behaviour and their influence on ageing, to find materials that best meet the specifications needed to reduce ghost fishing through new gear development.

## Data Availability

The datasets used and/or analysed during the current study will be available from the corresponding author upon reasonable request.

## Code availability

The Python code used for the analysis of the different scales can be found here: https://github.com/llegue/mkf-app.
